# The Effect of Response Inhibition Training on Risky Decision-Making Task Performance

**DOI:** 10.3389/fpsyg.2020.01806

**Published:** 2020-07-24

**Authors:** Pengbo Xu, Di Wu, Yuqin Chen, Ziwei Wang, Wei Xiao

**Affiliations:** ^1^Department of Medical Psychology, Air Force Medical University, Xi’an, China; ^2^Second Brigade, NCO School, Army Medical University, Shijiazhuang, China

**Keywords:** response inhibition, risky decision-making, Balloon Analog Risk Task, Go/NoGo, stop-signal task

## Abstract

Response inhibition is an important component of executive function and plays an indispensable role in decision-making and other advanced cognitive processes. At the same time, we need an effective way to improve decision-making in the face of complex and limited information. This study mainly explored the influence of response inhibition training on college students’ risky decision-making. The recruited students were randomly divided into the training group (*n* = 28) and the control group (*n* = 28). The training group engaged in Go/NoGo and stop-signal tasks for 2 weeks, while the control group was given the task of reading and summarizing popular science articles related to self-control. The Stroop task and Balloon Analog Risk Task were used to evaluate the pretest and posttest performance in inhibitory control and risky decision-making tasks, respectively, for all subjects. The results showed that response inhibition training can be effectively transferred to interference control task performance. The results showed that both the reward acquired and adjusted Balloon Analog Risk Task score (adj BART) significantly improved compared to the pretest in the training group, while the control group showed no significant differences in the reward acquired and the adj BART between the pretest and the posttest. Although response inhibition training increased risky behaviors in the Balloon Analog Risk Task, it substantially reduced overly conservative behaviors and participants gained more money.

## Introduction

There are all kinds of risky decisions that we make in life. From daily shopping to financial investments, people always need to make choices with limited time and information resources. Many studies have shown that people’s decision-making is influenced by gender, individual characteristics, emotional states, cognitive abilities, irrelevant information, and so on (Sun et al., 2015; Stanovich, 2018; Talukdar et al., 2018; Weller et al., 2018). However, little is known about whether a simple and an operational training method can effectively affect decision-making. In the dual-process theories of decision-making, System 1 processes are often automatic, fast, and easily affected by emotion, while System 2 processes are relatively slow and rational process, in which the most important function of System 2 is the successful override of System 1 (Stanovich and West, 2008; Evans and Stanovich, 2013; Stanovich, 2018). Moreover, the selection of alternative responses in the decision-making process also depends on the continuous effectiveness of regulatory control processes (Moeller et al., 2001). That is, to achieve better decision-making, it is necessary to continuously control their own dominant responses and irrelevant interference information, which is undoubtedly closely related to the ability of inhibitory control. The purpose of this paper is to determine whether primary cognitive training can effectively change high-level decision-making behavior. If the method of improving risky decision-making ability through primary cognitive training (such as inhibitory control) is proven and widely accepted, it will greatly advance the research process in the field of decision-making and will certainly provide a direction for future development.

Inhibitory control, one of the important components of executive function, is the ability to suppress irrelevant, interfering, incorrect or inappropriate goal-directed dominant responses, impulses, behavioral choices, and automatic behavioral habits (Barkley, 1997; Miyake et al., 2000; Munakata et al., 2011; Enge et al., 2014). Inhibitory control can be roughly divided into reaction inhibition and interference control; the former mainly focuses on the suppression of the dominant response, while the latter focuses on the suppression of irrelevant information. Many studies have shown that inhibitory control plays an important role in verbal communication (Bishop and Norbury, 2005), reading comprehension (Wang and Gathercole, 2015; Potocki et al., 2017), memory retrieval (Depue et al., 2010; Penolazzi et al., 2014), and mathematical ability (Gilmore et al., 2013) and is involved in other higher cognitive processes, such as problem-solving and decision-making (Sakagami et al., 2006; Shenoy and Yu, 2011). In addition, inhibitory control training can affect working memory and fluid intelligence (Liu et al., 2015), and there have already been some practical applications in controlling addictive behavior, losing weight, reducing diet consumption, and improving mental illness (Houben, 2011; Bartholdy et al., 2016). Therefore, inhibitory control can be considered a basic ability that people must have.

The traditional view is that inhibitory control is an internal top-down execution process (Aron et al., 2004). An increasing amount of research results shows that top-down implementation of frontal regions is not always necessary to inhibit control behaviors and the participation of these inhibitory regions can be automatically driven by specific stimuli (Lenartowicz et al., 2011). Manuel et al. compared event-related potentials (ERPs) before and after auditory Go/NoGo task training and found decreased activity in the left parietal cortex (Manuel et al., 2010), suggesting that the repeated and stable association between the stimulus and inhibition response in the Go/NoGo task resulted in the gradual separation of top-down connectivity in the frontal lobe, thus facilitating rapid automatic inhibition. This is also the purpose of inhibitory control training, that is, training the slow thought suppression process into an automated, faster process.

There are many disputes about whether there is a close relationship between performance in the inhibitory control task and the risky decision-making task. Kertzman et al. (2018) used the performance on the Go/NoGo task, the Matching Familiar Figures Test (MFFT), and the Stroop task as indicator of inhibition ability and used the Iowa task performance as an indicator of risk and found no direct correlation between the two (Kertzman et al., 2018). They suggested that although there is some overlap between inhibitory control and the cognitive processing of risky decision-making, they may represent two relatively independent abilities. This may have something to do with the limitations of the Iowa gambling task itself. Previous studies have demonstrated that short-term stop-signal task (SST) training can reduce risk-taking behavior in gambling tasks (Verbruggen et al., 2012; Stevens et al., 2015). They designed a special training study and systematically studied the generalization model of promoting automatic inhibition and developing a top-down control inhibition training program. Simply training people to control their exercise behavior induced them to make cautious and risk-averse decisions for at least 2 h and the effect was comparable to that found in previous studies that used transcranial direct current stimulation (tDCS) to control risk (Fecteau et al., 2007).

The previous studies examined only immediate changes after training and the decision task selection typically included a single task. On the basis of previous studies, we chose the Balloon Analog Risk Task that has strong operability with initial results that are relatively stable and we appropriately increased the time interval between cognitive training and posttest decision-making task assessment. After excluding the immediate effects of training, we wanted to demonstrate that classic response inhibition training can also effectively change performance in this task. It can make the response suppression training more stable, more generalizable, and more convincing for improvements in risky decision-making. This paper proposes a hypothesis: classic response inhibition training can reduce risk behavior in the balloon simulation risk task, thereby resulting in more rewards.

## Materials and Methods

### Participants

A total of 56 university students were randomly divided into a training group (18 men and 10 women; mean age = 19.54 years, *SD* = 1.20) and a control group (18 men and 10 women; mean age = 19.46 years, *SD* = 1.55). There was no significant difference in age between the two groups [*t* (54) = 0.193, *p* = 0.848]. Subjects were included based on the following inclusion criteria: they were 18–22 years old, physically and mentally healthy, right-handed, with normal vision or corrected-to-normal vision, and had not participated in other relevant psychological experiments. The exclusion criteria were as follows: neurological disorders, alcohol or other substance abuse or overdependence, mental illness, and treatment with any psychotropic substance. The training process in our experiment is all person-by-person training. Under the condition of limited manpower and financial resources, the workload of these subjects is close to the maximum. However, *post hoc* power calculation is calculated by GPower 3.1.9.2 with a sample size of 56 participants, a significance level of 5% and an effect size of 0.2. The calculated power value is 0.95, proving that the sample size is sufficient. This research was approved and strictly implemented the recommendations of the Local Ethics Committee. All subjects were given detailed experimental instructions and agreed to participate in the experiment. Then, they signed the informed consent in accordance with the Declaration of Helsinki and were entitled to a certain payment after the experiment was completed.

### Training Session

The training group adopted the Go/NoGo task and SST as training tasks that were presented using E-prime 3.0. All experiment started with a short practice phase to make sure that the subjects understood the rules of the task completely. All training tasks were completed in the laboratory over a total of 4 weeks. The two groups were assessed with the Stroop task and the Balloon Analog Risk Task to evaluate the pretest and posttest performance on inhibitory control and risky decision-making tasks at weeks 1 and 4. In the intervening 2 weeks in the training group, two classic paradigms were simultaneously used and cross trained. The whole training schedule consisted of 30-min sessions, three times per week. During each training session, the two tasks alternated twice. In this study, two kinds of response inhibition training tasks were adopted. On the one hand, to increase the generalization effect of training, the inhibitory control ability was improved by automatic inhibition and top-down control inhibition (Spierer et al., 2013). On the other hand, with this kind of pure training, the more obvious the training boost will be and the more likely that the change in gambling task performance was due to improved inhibitory control. The control group read popular science articles related to self-control and were required to complete a task of summarizing the articles. The time and frequency for this task were consistent with the cognitive training of the training group.

#### Go/NoGo

There were double triangles (“Go”) and single triangles (“NoGo”) used as stimuli in the task. The participants had to make a button-press response to the double triangle and inhibit their response to the single triangle. Go and NoGo stimuli were randomly presented in a 3:2 ratio. All stimuli were presented for 100 ms in the center of the screen with a 1,200 ms inter-stimulus interval. The experimental phase consisted of Go stimuli of 240 trials and NoGo stimuli of 160 trials. There was a pause at the halfway point of this task and the participants could take a break or press any key to continue the experiment. The optimal performance of the task is to minimize the response time and the number of errors (the sum of the number of omission errors and commission errors). The task is the classic response inhibition task paradigm that has been widely used and is also recognized as a method that reflects inhibitory control abilities in a relatively simple and pure way (Congdon et al., 2012; Wostmann et al., 2013; Enge et al., 2014).

#### Stop-Signal Task

The participants were required to press the “f” or “j” key when the “f” or “j” letter (Go signal), respectively, appeared, and during a relatively low proportion of trials (30%) with obvious red dots (stop signal) appearing after the Go signal, to immediately suppress the impulse to press the button. The task consisted of 200 trials, including 120 no-stop stimuli and 60 stop stimuli. The center of the screen shows a fixation point (+) of 250 ms before all the stimuli appear, and there is a 1,000 ms interval after the button response. In the no-stop stimulus, the most presentation time of the Go signal was 1,250 ms. If the subject does not press the button in time, the screen will show “too slow”. In the stop stimulus, the stop signal will appear later than the Go signal and the stimulus presentation time is still 1,250 ms at most. If the subject does not immediately suppress the key, there will also be an interval of 1,000 ms. The difference between this task and the Go/NoGo task is that each stop signal is preceded by a reaction impulse and the clever experimental design allows a measure of behavioral inhibition time (Verbruggen and Logan, 2009; Verbruggen et al., 2019; i.e., stop-signal reaction time, SSRT). The tracking method was used to automatically adjust the time when the stop signal appeared (i.e., stop-signal delay, SSD) and the initial value was set at 250 ms. When the inhibition was successful, the SSD increased by 50 ms, while the inhibition failed, and the SSD decreased by 50 ms to ensure that the successful inhibition rate of the subjects was approximately equal to 50%. Then, the SSRT value can be calculated (SSRT was equal to the average Go reaction time minus the average SSD).

### Measures

#### Stroop Task

The Stroop task, which is commonly used for inhibitory control and relatively complicated in processing, was used to evaluate the ability of stimulus interference to inhibit or selectively focus on target-related stimuli (Turner et al., 2017). The participants were asked to select the corresponding key according to the four colors of red, blue, green, and yellow fonts. All stimuli were presented for 100 ms followed by fixation point (+) for 250 ms and there was a 1,000 ms interval after the subject responded. The task consisted of 160 trials. Four colors and four fonts were randomly matched and presented (every font was matched with four different colors, so that the ratio of consistent trials to inconsistent trials was 1:3) and consistent and inconsistent response times (RT) were recorded. The conflict effect (incongruent trials RT – congruent trials RT) and conflict score (conflict effect/congruent trials RT) were calculated to evaluate the two groups before and after the inhibitory control ability training (Maraver et al., 2016).

#### Balloon Analog Risk Task

The Balloon Analog Risk Task was used to evaluate risky behaviors and is a decision-making task that can effectively simulate realistic risky behaviors that are relatively stable under laboratory conditions (Lejuez et al., 2002). The experimental programming of this task was based on computer programming (C++) prepared and rendered on the computer screen. The participants can make money by inflating the balloon with a click of the mouse (earning 2 yuan per inflation, which was included in the temporary account), but if the balloon bursts, they lose the money they made during the round (the temporary account). At the same time, the participants can choose to stop the pump at any time and the temporary account is transferred into the permanent account. Each balloon is blown between 1 and 32 times and there is a predetermined explosion point (randomly set by the computer). The participants were asked to conduct 30 balloon trials to make money and were given the sum from their permanent accounts for the 30 balloon trials. The only way to make money is to stop the balloon before it explodes. The subjects were also told that the goal was to inflate the balloon as large as possible without exploding to maximize the benefit. The final benefit of each subject was recorded and average adjusted pumps (i.e., adj BART; adj BART = total number of unexploded balloon pumps/number of unexploded balloons) was calculated to measure the performance and impulsivity in the task.

### Data Analysis

First, a curve was drawn between the performance in the two tasks in the training group and the training time. Then, two independent sample *t*-tests were conducted on the pretest values of the Stroop task and Balloon Analog Risk Task for the two groups and no significant difference was found between the two groups at pretest. Because the experiment adopted a mixed design with between‐ and within-subjects factors, mixed-model ANOVAs of 2 (control group and training group) × 2 (pretest and posttest) factors were used to evaluate the transfer effect of response inhibition training to Stroop performance and its impact on Balloon Analog Risk Task performance. Finally, we further analyzed the correlation between the initial threshold and the change amount of the training group.

## Results

### Training Results

**Figure 1:**
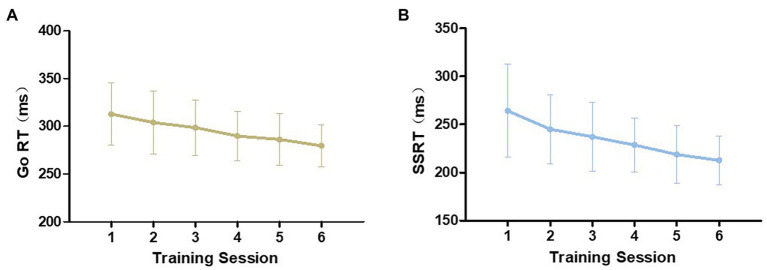
Training improvement during the six training sessions in the training group. Mean values and standard errors of Go reaction times in Go/NoGo task **(A)** and SSRT in stop-signal task **(B)** are visualized.

**Figure 2:**
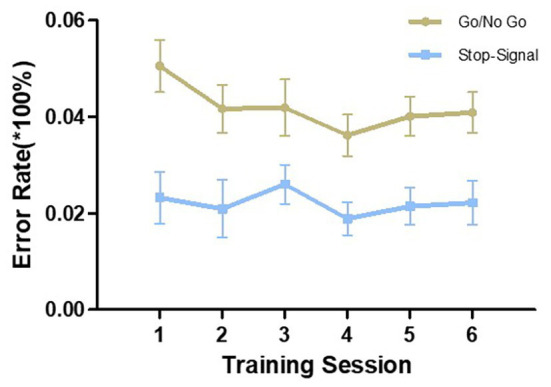
Error rate in the two training tasks during the six training sessions. The error in the Go/NoGo task includes omission errors and commission errors. The error in the stop-signal task refers to errors in the Go response and does not include the number of suppression failures. Error bars indicate standard errors of the mean.

Since both tasks were completed twice in one training session, we took the average of the two as the performance for that training session. Repeated-measures ANOVAs were conducted to compare the performance in the first session with that of the sixth session. As shown in Figure 1, both Go RT and SSRT were gradually reduced from the first to the last training session in their respective tasks and the differences reached statistical significance [Go RT: *F* (1,27) = 34.987, *p* < 0.001, *η*^2^ = 0.564; SSRT: *F* (1,27) = 38.416, *p* < 0.001, *η*^2^ = 0.587]. In the two tasks, the error rate did not significantly change from the first to the sixth training session [Go/NoGo task: *F* (1,27) = 3.057, *p* = 0.092, *η*^2^ = 0.102; SST: *F* (1,27) = 0.028, *p* = 0.869, *η*^2^ = 0.001; Figure 2]. However, there was a downward trend in the Go/NoGo task, especially across the first four training sessions.

### Stroop Task

**Figure 3:**
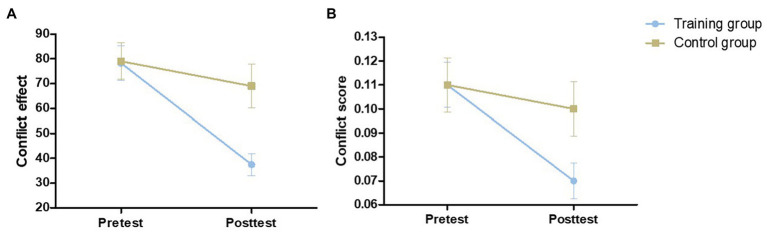
The performance of the two groups on the Stroop at pretest and posttest. Conflict effect **(A)** = incongruent trials RT – congruent trials RT. Conflict score **(B)** = conflict effect/congruent trials RT. Error bars indicate standard errors of the mean.

The pretest conflict effect [*t* (54) = 0.075, *p* = 0.941] and conflict score [*t* (54) = 0.111, *p* = 0.912] were not significantly different between the control group and training group. We assessed the effect of training using mixed-model ANOVAs with test time and group as within‐ and between-subject factors, respectively. The main effect of group on the conflict effect was not significant [*F* (1,54) = 3.971, *p* = 0.051, *η*^2^ = 0.068], and the main effect of group on the conflict score was also not significant [*F* (1,54) = 2.127, *p* = 0.151, *η*^2^ = 0.038]. The main effect of testing time was significant [conflict effect: *F* (1,54) = 18.622, *p* < 0.001, *η*^2^ = 0.256; conflict score: *F* (1,54) = 14.397, *p* < 0.001, *η*^2^ = 0.210]. As shown in Figure 3, we also found a significant time × group interaction effect [conflict effect: *F* (1,54) = 6.821, *p* = 0.012, *η*^2^ = 0.112; conflict score: *F* (1,54) = 5.664, *p* = 0.021, *η*^2^ = 0.095]. Through simple effect analysis, two groups of effects were obtained. The conflict effect and conflict score in the training group after training were significantly lower (*p* < 0.001), while no significant difference between pretest and posttest performance was found in the control group [conflict effect: *F* (1,27) = 1.025, *p* = 0.320, *η*^2^ = 0.037; conflict score: *F* (1,27) = 0.697, *p* = 0.411, *η*^2^ = 0.025; Table 1]. The inhibitory control ability in the training group was improved compared with that of the control group because of the training.

**Table 1:** Group mean (±*SD*) pretest and posttest scores on the Stroop task and the Balloon Analog Risk Task.

	Training group (*n* = 28)				Control group (*n* = 28)			
	Pretest	Posttest	*d*	*p*	Pretest	Posttest	*d*	*p*
Stroop task								
Conflict effect	78.32 ± 36.89	37.45 ± 23.52	0.60	<0.001^∗^	79.07 ± 38.87	69.02 ± 47.00	0.04	0.320
Conflict score	0.11 ± 0.05	0.07 ± 0.04	0.56	<0.001^∗^	0.11 ± 0.06	0.10 ± 0.06	0.03	0.411
Errors	11.25 ± 8.04	12.21 ± 9.53	−0.02	0.470	11.89 ± 7.00	12.54 ± 8.84	−0.01	0.609
Balloon Analog Risk Task								
Adj BART	11.87 ± 3.31	14.06 ± 2.65	0.43	<0.001^∗^	11.91 ± 3.94	12.15 ± 2.92	0.01	0.648
Reward	363.64 ± 87.67	430.14 ± 71.98	0.30	0.002^∗^	382.00 ± 89.90	389.79 ± 92.19	0.01	0.680

Significance (*p*) and effect sizes (Cohen’s *d*) are reported for the repeated-measures ANOVAs as a within-subject variable (pretest and posttest). ^*^p < 0.01.

### Balloon Analog Risk Task

**Figure 4:**
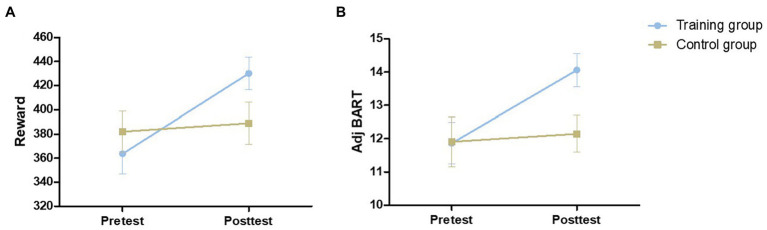
The performance of the two groups on the Balloon Analog Risk Task at pretest and posttest. Reward is the sum of permanent accounts for the 30 balloon trials **(A)**. Adj BART (average adjusted pumps) = total number of unexploded balloon pumps/number of unexploded balloons **(B)**. Error bars indicate standard errors of the mean.

**Figure 5:**
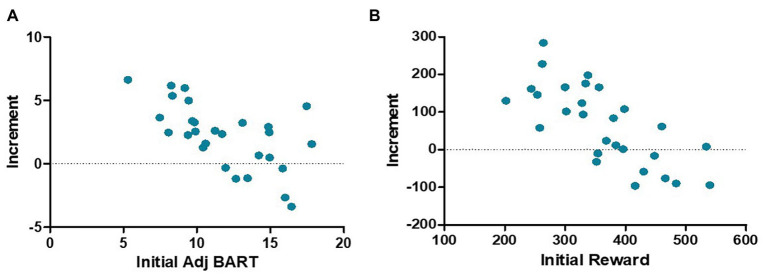
A scatter plot of increment and initial thresholds in the Balloon Analog Risk Task. For both the adj BART **(A)** and reward **(B)**, there is a significant negative correlation between the initial value and the increment.

As shown in Figure 4, there was no significant difference between the two groups in the pretest adj BART [*t* (54) = 0.046, *p* = 0.964] or the reward obtained in the Balloon Analog Risk Task [*t* (54) = 0.774, *p* = 0.443]. The 2 (control group and training group) × 2 (pretest and posttest) mixed-model ANOVAs were performed for the reward and adj BART in the two groups: no significant main effect of group [reward: *F* (1,54) = 0.355, *p* = 0.554, *η*^2^ = 0.007; adj BART: *F* (1,54) = 1.389, *p* = 0.244, *η*^2^ = 0.025] was found. The main effect of time was significant [reward: *F* (1,54) = 7.488, *p* = 0.009, *η*^2^ = 0.121; adj BART: *F* (1,54) = 11.788, *p* = 0.001, *η*^2^ = 0.179]. The interaction of the group and time was also obviously significant [reward: *F* (1,54) = 4.653, *p* = 0.035, *η*^2^ = 0.079; adj BART: *F* (1,54) = 7.615, *p* = 0.008, *η*^2^ = 0.124]. Then, the effects of the two factors were assessed through simple effect analysis. Both the reward acquired and adj BART for the training group were significantly increased in the posttest compared with the pretest [reward increased by 66.50 yuan on average, *F* (1,27) = 11.273, *p* = 0.002, *η*^2^ = 0.295; adj BART increased 2.56 times on average, *F* (1,27) = 20.554, *p* < 0.001, *η*^2^ = 0.432], while there was no significant difference between the pretest and posttest measures for the control group [reward: *F* (1,27) = 0.174, *p* = 0.680, *η*^2^ = 0.006; adj BART: *F* (1,27) = 0.213, *p* = 0.648, *η*^2^ = 0.008]. We further analyzed the correlation between the initial threshold and the change in the training group and found a significant negative correlation (reward: *r* = −0.734, *p* < 0.001; adj BART: *r* = −0.620, *p* < 0.001; Figure 5). The results indicated that adj BART and reward acquired by the training group after training significantly increased compared with that before training. Moreover, the subjects with lower pretest indexes had a greater range of changes through training.

## Discussion

From the results of the whole experiment, we not only improved the performance of the training task through 2 weeks of response inhibition training (Go/NoGo and stop-signal tasks), but also more importantly, we found the migration effect of training in the untrained Stroop task and Balloon Analog Risk Task. The fact that inhibition control plays an important role in people’s complex decision-making process has been verified.

There was a gradual improvement in the performance of the training group in two classic response inhibition tasks. The error rate in the Go/NoGo task (the ratio of the sum of omission errors and commission errors) showed a downward trend over the first four training sessions and increased in the later sessions. Moreover, it can be seen from the results of the last two training sessions that the task response decreased while the error rate increased. This may have been because the participants were too reactive, which sometimes led to a rebound in error rates. In the later sessions, there was a gradual balance between the reaction time and the error number, and finally, it tended to be stable. Therefore, although the error rate during the last four training sessions showed a slight upward trend, the overall response time showed a downward trend and an obvious training effect could still be seen. The error rate in the SST refers to the proportion of errors in the Go response, not the proportion of suppression failures. The low error rate during the first exposure may have been due to the relatively small allocation of cognitive resources in the process of inhibition. With the increase in the allocation of cognitive resources in the inhibition process, the accuracy of keystrokes is ignored and the error rate changes little or slightly increases. However, we can still see the improvement in task performance from the trend in the SSRT scores. There was a significant difference in the effect of the two kinds of training, and it was also found that the pursuit of reaction speed might lead to a decrease in accuracy in the later periods of training, and eventually, the two tended to stabilize. In addition, Enge et al. also found that in the latter stage of training in the SST, SSD and mean Go reaction time were simultaneously reduced, which would eventually lead to the reverse increase in SSRT (Enge et al., 2014). In this experiment, this phenomenon occurred in some subjects, but the overall trend was not found.

Lower conflict scores in the Stroop task in the training group after training suggested that response inhibition training could be transferred to interference control. This also showed that the two classic response inhibition task training methods are effective. Friedman and Miyake used latent variable analysis to demonstrate that almost all inhibition tasks have a common inhibitory control mechanism (Friedman and Miyake, 2004). Although Brydges et al. (2012) later used ERP technology to show that the two tasks engaged two different cognitive components (Brydges et al., 2012), they were still closely related, and performance could be transferred (Maraver et al., 2016).

From the adj BART in the Balloon Analog Risk Task, it can be concluded that the training of response inhibition led to an increase in the subjects’ risky behaviors, which seems to contradict our hypothesis. However, at the same time, the reward acquired in the task increased after the training. According to the value gained by pumping up the balloon and the probability of explosion, the value of the first 16 rounds of pumping is greater than the value of the nonpumping rounds. It is not until the seventeenth turn that pumping up the balloon becomes irrational (Lejuez et al., 2002). Therefore, from the perspective of profits obtained, the subjects were too conservative to avoid balloon explosion, thereby losing the chance to win more money before training. This also explained our increased risk-taking behavior and benefits after training. We therefore suggest that the key decision for Balloon Analog Risk Task is not to inflate (this is a continuous process) but to decide when to stop inflating and put the contents of the temporary account into the permanent account. Only by making a rational decision to stop inflating (properly suppressing decisions that are too early or too late) can the maximal amount of reward be obtained. Each inflation is actually equivalent to a Go reaction, and it is the ability of response inhibition that is needed as the basis for the critical and appropriate stopping of inflation. The results of increasing risk-taking behaviors in this paper were inconsistent with those of the previous study (Verbruggen et al., 2012). Although many gambling tasks are task paradigms for evaluating risky decision-making, different tasks represent different risky decision-making processes (Buelow and Blaine, 2015). The stopping of inflation in the Balloon Analog Risk Task may be more related to the ability to response inhibition, which may also have contributed to the inconsistent results across different tasks.

We further analyzed the significant negative correlation between the initial threshold and the change amount in the training group and found that the lower the initial value was, the more significant the improvement. This suggested, to some extent, that people with lower initial values were more likely to improve (Schmaal et al., 2013). Therefore, we should probably focus more on the lower level of the population, where the limited training intensity could achieve a higher training effect. There are many other factors that affect inhibitory control training. It is widely recognized that emotion and motivation affect cognitive inhibition processes, higher decision-making processes, and other neural or psychological functions (Padmala and Pessoa, 2011; Turner et al., 2018). In the traditional sense, it is considered that subcortical structures, such as the amygdala, ventral striatum, and hypothalamus, are mainly responsible for processing emotions and behaviors, while cortical structures, such as the dorsolateral prefrontal cortex and anterior cingulate cortex, are responsible for activating cognitive control and higher executive functions (Pessoa, 2008). Therefore, appropriate incentives and positive feedback will effectively improve the training effect of the subjects. In addition, some studies failed to achieve transfer effects of training (Enge et al., 2014; Zhao et al., 2015; Kable et al., 2017), which may be because the differences were not significant due to the insufficient number of subjects or the training time and intensity did not reach the threshold needed to transfer tasks (Kable et al., 2017). As shown in Figure 4, there were also some subjects with opposite results, but this pattern of results were mainly concentrated in those with higher starting values. If the primary group chosen were primarily high-level people, then group-level indifference would be inevitable. Therefore, the initial level of grouping will also affect the training effect.

The basis for improving decision-making through training in response inhibition is brain plasticity (i.e., a change in behavior and its underlying brain anatomy based on experience; Spierer et al., 2013). These changes can facilitate the acquisition of new skills, the improvement of acquired abilities, and the recovery of defective or impaired functions (Kelly and Garavan, 2005). Changes in behavior and brain plasticity induced by training have been demonstrated at different levels of executive function. Research has shown that people’s inhibitory control ability is closely related to the inferior frontal gyrus and dorsolateral prefrontal cortex (Aron et al., 2004). At the same time, these brain regions also play an important role in risky decision-making tasks (Chiu et al., 2012). During the training of the inhibitory control tasks, the corresponding brain regions will be repeatedly activated and the connections between the corresponding brain regions will be increased, which will inevitably affect the neural connections in the decision-making process. Although there is no neuroscientific evidence, it is likely that this is one of the important reasons that response inhibition training changes subsequent performance in decision-making tasks. The results of the control group also showed that reading about self-control skills alone was not enough to improve the participants’ inhibitory control skills, which also reflected the need for cognitive training of response inhibition.

## Limitations

First, the selection range of the subjects was relatively limited, leading to limited generalization. Second, the training time was short, and there was no long-term tracking due to the effect of novel coronavirus, so the duration of the transfer effect cannot be determined at present. Third, the degree of improvement in the Balloon Analog Risk Task performance in the training group was relatively limited, which may also be related to shorter training time and lower intensity. Fourth, as the training group needs to spend a lot of time in the whole training process, our subjects may be potentially inadequate. The problem of subject size is also a shortcoming of most cognitive training studies. Because of this, slight differences in some experimental conditions may lead to different migration outcomes or no migration effects between many similar training studies. In addition, for the measurement of inhibitory control and risky decision-making, a variety of evaluation indicators should be used, such as questionnaires, behavioral observation, and imaging techniques such as ERPs and magnetic resonance imaging (MRI), rather than cognitive task paradigms on computers. A single score from the inhibition task or risky decision-making task cannot represent the complex control processes that may be correlated with each other, so it is necessary to use multiple tasks to evaluate the inhibitory control ability and risky decision-making (Dougherty et al., 2005).

## Conclusion

This study confirms that classic response inhibition training can increase risk-taking behavior in the Balloon Analog Risk Task, improve their overly conservative behaviors, properly inhibit them to obtain more benefits, and substantially increase economic rationality. During the whole experiment, various experimental conditions were strictly controlled and the training and transfer effects were statistically significant. It is particularly important that compared with inhibiting the near transfer between control tasks, the risky decision-making task can be considered a far transfer (Crespi et al., 2018) and this experiment is a good attempt at selecting a cognitive training far transfer task. Research on the transfer effect of inhibitory control training to higher cognitive function and the tracking of training will also become the focus of future research in this field. However, at the same time, we must also make it clear that these higher cognitive processes are not just inhibitory control processes, and whether there is a more general, fundamental process is debatable.

## Data Availability Statement

The raw data supporting the conclusions of this article will be made available by the authors, without undue reservation.

## Ethics Statement

The studies involving human participants were reviewed and approved by Air Force Medical University. The patients/participants provided their written informed consent to participate in this study.

## Author Contributions

PX completed the experiment and wrote it. DW and YC assisted the experiment and analyzed the data. ZW provided technical guidance and site support. WX grasped the idea and financial support. All authors contributed to the article and approved the submitted version.

### Conflict of Interest

The authors declare that the research was conducted in the absence of any commercial or financial relationships that could be construed as a potential conflict of interest.
